# Features That Middle-aged and Older Cancer Survivors Want in Web-Based Healthy Lifestyle Interventions: Qualitative Descriptive Study

**DOI:** 10.2196/26226

**Published:** 2021-10-06

**Authors:** Nataliya V Ivankova, Laura Q Rogers, Ivan I Herbey, Michelle Y Martin, Maria Pisu, Dorothy Pekmezi, Lieu Thompson, Yu-Mei M Schoenberger-Godwin, Robert A Oster, Kevin Fontaine, Jami L Anderson, Kelly Kenzik, David Farrell, Wendy Demark-Wahnefried

**Affiliations:** 1 Department of Health Services Administration University of Alabama at Birmingham Birmingham, AL United States; 2 Division of Preventive Medicine University of Alabama at Birmingham Birmingham, AL United States; 3 Department of Surgery University of Alabama at Birmingham Birmingham, AL United States; 4 Health Science Center University of Tennessee Memphis, TN United States; 5 Department of Health Behavior University of Alabama at Birmingham Birmingham, AL United States; 6 Division of Hematology & Oncology University of Alabama at Birmingham Birmingham, AL United States; 7 People Designs Durham, NC United States; 8 Department of Nutrition Sciences University of Alabama at Birmingham Birmingham, AL United States

**Keywords:** cancer survivors, diet, physical activity, lifestyle, internet, interventions, qualitative, eHealth, mobile phone

## Abstract

**Background:**

With the increasing number of older cancer survivors, it is imperative to optimize the reach of interventions that promote healthy lifestyles. Web-based delivery holds promise for increasing the reach of such interventions with the rapid increase in internet use among older adults. However, few studies have explored the views of middle-aged and older cancer survivors on this approach and potential variations in these views by gender or rural and urban residence.

**Objective:**

The aim of this study was to explore the views of middle-aged and older cancer survivors regarding the features of web-based healthy lifestyle programs to inform the development of a web-based diet and exercise intervention.

**Methods:**

Using a qualitative descriptive approach, we conducted 10 focus groups with 57 cancer survivors recruited from hospital cancer registries in 1 southeastern US state. Data were analyzed using inductive thematic and content analyses with NVivo (version 12.5, QSR International).

**Results:**

A total of 29 male and 28 female urban and rural dwelling Black and White survivors, with a mean age of 65 (SD 8.27) years, shared their views about a web-based healthy lifestyle program for cancer survivors. Five themes emerged related to program content, design, delivery, participation, technology training, and receiving feedback. Cancer survivors felt that web-based healthy lifestyle programs for cancer survivors must deliver credible, high-quality, and individually tailored information, as recommended by health care professionals or content experts. Urban survivors were more concerned about information reliability, whereas women were more likely to trust physicians’ recommendations. Male and rural survivors wanted information to be tailored to the cancer type and age group. Privacy, usability, interaction frequency, and session length were important factors for engaging cancer survivors with a web-based program. Female and rural participants liked the interactive nature and visual appeal of the e-learning sessions. Learning from experts, an attractive design, flexible schedule, and opportunity to interact with other cancer survivors in Facebook closed groups emerged as factors promoting program participation. Low computer literacy, lack of experience with web program features, and concerns about Facebook group privacy were important concerns influencing cancer survivors’ potential participation. Participants noted the importance of technology training, preferring individualized help to standardized computer classes. More rural cancer survivors acknowledged the need to learn how to use computers. The receipt of regular feedback about progress was noted as encouragement toward goal achievement, whereas women were particularly interested in receiving immediate feedback to stay motivated.

**Conclusions:**

Important considerations for designing web-based healthy lifestyle interventions for middle-aged and older cancer survivors include program quality, participants’ privacy, ease of use, attractive design, and the prominent role of health care providers and content experts. Cancer survivors’ preferences based on gender and residence should be considered to promote program participation.

## Introduction

### Background

Over 16 million individuals in the United States are living with a history of cancer, a prevalence expected to grow to over 22 million by 2030 [1]. The risk of cancer increases with age; thus, cancer survivors aged ≥65 years are anticipated to comprise approximately 73% and aged 50 to 64 years approximately 18% of the survivors by 2040 [2]. Cancer survivors are at a greater risk of cancer recurrence or second malignancy [3], and accelerated aging [4], which increases mortality risk [5]. Healthy eating, physical activity, and weight management can attenuate these health risks and functional decline [6,7]; however, only 29% of cancer survivors have normal weight, 27% eat at least 5 daily servings of vegetables and fruit, and 47% engage in at least 150 minutes per week of aerobic physical activity (only 34% for older cancer survivors) [8,9].

Technology offers several important advantages for health behavior change interventions, such as increased access, greater user convenience, lower user cost, and personalized tailoring [10-13]. Internet use is rapidly increasing among adults aged ≥50 years, who represent the majority of cancer survivors [1,2]. About 88% of US adults aged 50 to 64 years and 73% aged ≥65 years are internet users, with the most rapid increase in use among adults aged ≥65 years (ie, from 57% in 2014 to 73% in 2019) [14]. Identifying features that promote participation in technology-based lifestyle interventions may support the realization of these potential advantages. Prior research indicates that cancer survivors prefer web-based health care technology and interventions (known as eHealth) [15], if the intervention provides tailored survivorship care plans, education to prevent cancer recurrence, and communication with fellow cancer survivors [16,17]. Although many middle-aged and older adults perceive the electronic exchange of health information as important [18], few studies have included middle-aged and older cancer survivors—a subgroup not often targeted specifically in eHealth literature. Moreover, studies rarely report variations in survivors’ preferences based on gender and geographic location (rural and urban) [19].

### Objective

The aim of this study was to explore the views of middle-aged and older cancer survivors regarding features of web-based healthy lifestyle programs to inform the development of a web-based diet and exercise intervention. We included cancer survivors aged ≥65 years while also reflecting the perspectives of cancer survivors who are aging into the group within the next 10 to 15 years. In addition, we wanted to capture potential variations in these views by gender and rural and urban status.

## Methods

### Design

We used a qualitative descriptive approach [20] to explore the perspectives of a diverse sample of cancer survivors on the design of a web-based healthy lifestyle intervention. According to the Rogers' Diffusion of Innovation (DOI) Theory, the characteristics of innovation are crucial to its adoption and use [21]. Therefore, we considered it important to use a pragmatic perspective to explore the characteristics of innovation through the views of its potential users. A qualitative descriptive approach allows data interpretation that closely reflects participants’ views and aims to uncover individuals’ perspectives on the studied phenomenon [22]. It also allows the research results to emerge from the data without undue restraints of a structured approach [23]. The study protocol was approved by the University of Alabama at Birmingham and the University of Tennessee Health Science Center Institutional Review Boards.

### Participants

Using a purposeful sampling strategy [24], cancer survivors were recruited from a hospital tumor registry in a southeastern US state using recruitment letters followed by a screening telephone call. The goal was to recruit the best informants [24], who would provide insightful views related to design and participation in the internet program based on their cancer survivor experience. Eligibility criteria included adults who (1) were aged ≥45 years; (2) were diagnosed within 1 to 5 years with a localized cancer of the breast, colorectum, endometrium, ovary, genitourinary (prostate), kidney, or multiple myeloma; (3) were English-speaking; (4) were community dwelling; (5) completed eighth grade or higher; (6) had BMI of at least 25 kg/m^2^ but less than 50 kg/m^2^; (7) do not engage in regular exercise; and (8) eat <2.5 servings of fruits and vegetables per day. In addition, the opportunity was advertised through cancer support groups and cancer types other than those in (2) were allowed if participants were from rural areas or Black survivors to maximize their representation. Potential participants were not screened for computer, smartphone, or mobile phone access at the time of recruitment.

### Data Collection

A total of 10 focus groups were conducted with 57 cancer survivors, with persons per focus group ranging from 2 to 12. Focus groups are effective for exploring potential users’ perspectives to inform intervention development [25,26]. To capture variations in survivors’ views, focus groups were both gender homogenous and mixed and were conducted in rural and urban areas [27] (Table 1). Rural and urban status was defined based on participants’ zip codes and the 2010 Urban Area to ZIP Code Tabulation Area Relationship File [28]. At the beginning of each focus group, we obtained informed consent; then participants completed a survey about their use of the internet, computers, and cell phones. To protect cancer survivors’ anonymity, each participant selected an alias to use during the discussion.

**Table 1:** Focus group composition.

Gender	Urban		Rural		Total focus groups (n=10), n (%)	Total participants (n=57), n (%)
	Focus groups, N	Participants, n (%)	Focus groups, N	Participants, n (%)		
Women	1	8 (21)	1	2 (11)	2 (20)	10 (18)
Men	3	12 (32)	1	4 (21)	4 (40)	16 (28)
Mixed	2	18 (47; n=9 women; n=9 men)	2	13 (68; n=9 women; n=4 men)	4 (40)	31 (54)

Considering one of the premises of Rogers' DOI Theory that the characteristics of innovation are essential for its potential adoption [21], the research team developed a focus group guide aimed to inductively generate information [20,23] related to cancer survivors’ use of eHealth: familiarity and use of healthy lifestyle websites providing information on diet and physical activity, cancer survivors’ preferences for learning and using technology, and type and frequency of feedback for participation in the program activities (Textbox 1). We also demonstrated and asked feedback on 3 web-based program features that were under consideration for a web-based program at that time: live web chat, Facebook discussion group, and Articulate Storyline interactive e-learning sessions. We chose these features because of their potential to facilitate engagement with a program, provision of social support, and easy access via multiple devices (smartphones and computers) [29-32]. Each feature was explained and demonstrated for focus group participants, followed by probing questions about the feature’s perceived effectiveness for delivering program content and promoting cancer survivors’ program participation. We also explored comfort levels with sharing information using these features (particularly Facebook discussion groups) and participants’ preferences for the duration and frequency of using these features.

**Sample focus group questions.**

**Sample focus group questions**

1. What health websites have you used for information on eating healthy and physical activity? What features did you like and dislike and find helpful and less helpful and why?
2. Introduction, demonstration, and discussion of 3 internet program features (see probing questions below).
  - *Live web chat* involves watching an informational video on a health-related topic, such as healthy eating, which is delivered via a website. With a live web chat, cancer survivors can watch the video, type questions, and receive answers from a staff member after the video is over.
  - *The Facebook discussion group* is dedicated to a specific community or membership or subjects, such as health, diet, lifestyle, cooking, social issues, and more. For example, cancer survivors can use the discussion group to talk about losing weight and other health-related issues with other members.
  - Articulate Storyline (interactive, e-learning sessions) allows cancer survivors to interact with the information in a video. For example, the Storyline can ask the survivor about the type of cancer and treatment and then provide advice about exercise or healthy eating that is personalized to the survivors’ needs.
    Probing questions for every feature: What would cancer survivors like about this feature? Why?; What would cancer survivors not like about this feature? Why?; Why would cancer survivors find this feature engaging?; Why would cancer survivors not find this feature engaging?; How often would cancer survivors use this feature?; How comfortable would cancer survivors be to use this feature?; What other comments do you have about this feature?
3. How would cancer survivors prefer to learn about how to use the internet program and technology?
4. What feedback and how often would cancer survivors like to receive about their progress in an internet healthy lifestyle program? How can cancer survivors use this feedback?

The focus group guide was pilot-tested using a mock focus group of volunteer cancer survivors and research staff. The guide was further refined through an iterative approach to data collection and analysis [33] when transcripts were reviewed and analyzed soon after the focus group completion to inform and adapt probing questions. Focus groups were facilitated by 2 experienced moderators and lasted approximately 2 hours. All sessions were audio recorded. Participants were provided with light refreshments and US $25 compensation for their time and travel.

### Data Analysis

Focus group recordings were transcribed verbatim by a professional transcription company. Verified transcripts were independently analyzed by 3 researchers (NVI, IIH, and LT) using inductive thematic [34] and content analyses [35] with NVivo (version 12.5 Plus, QSR International). The analytical process involved several steps. First, the researchers independently coded the original transcripts by identifying key points and recurring subthemes and themes that were central to the areas of discussion within and across the focus groups. A constant comparative method [36] that involves iterative comparison of new information with coded data was used to guide the analysis. This inductive analytical process allowed us to identify common themes and subthemes that transcended all focus groups while capturing variations in cancer survivors’ perspectives on the discussed topics. The researchers reviewed the merged coding results after the analysis of each transcript to resolve coding discrepancies. They also regularly met with the rest of the research team to discuss emergent themes and refine the codebook. An intercoder agreement was established at a recommended 90% [37].

When the thematic analysis of all focus groups was completed and saturation in the data was achieved, the researchers performed content analysis on the generated themes and codes using the counts of text references in NVivo to systematically represent consistencies and variations in viewpoints across the focus groups based on participants’ gender and residence. This analysis also helped identify how the themes were interrelated and interconnected to describe cancer survivors’ varied views on a web-based healthy lifestyle program. Demographic and survey data were analyzed using descriptive statistics with SAS (version 9.4, SAS Institute).

## Results

### Description of the Participants

A total of 57 survivors of 6 different cancer types participated in the focus groups (Table 2). The mean age was 65 (SD 8.27) years, and both genders were evenly represented (29/57, 51% men and 28/57, 49% women). About two-thirds were urban dwelling (37/57, 65%) and more than half were White (32/57, 56%) survivors. Most of the participants had cell phones (56/57, 98%) or smartphones (46/57, 81%) and a computer with internet access (35/57, 61%). More than half of the participants used email (33/57, 58%) and text messaging (39/57, 68%) at least once a day (Table 3).

**Table 2:** Demographic characteristics of focus group participants (N=57).

Characteristics		Participants, n (%)
**Gender**		
	Male	29 (51)
	Female	28 (49)
**Age (years)**		
	47-64	25 (44)
	65-74	24 (42)
	≥75	8 (14)
**Race**		
	Black	23 (40)
	White	32 (56)
	Other	2 (4)
**Cancer type**		
	Breast	17 (30)
	Prostate	18 (32)
	Multiple myeloma	7 (12)
	Colorectal	5 (9)
	Gynecologic (ovarian or endometrium)	7 (12)
	Other	3 (5)
**Residency status**		
	Rural	19 (33)
	Urban	37 (65)
	Missing	1 (2)
**Marital status**		
	Married or lives with partner	38 (67)
	Divorced, separated, or widowed	19 (33)
**Education**		
	High school or less	19 (33)
	Some college	16 (28)
	College graduate	22 (39)
**Employment**		
	Employed	13 (23)
	Retired	28 (49)
	Homemaker	2 (4)
	Unable to work	7 (12)
	Other	7 (12)
**Household income level (US $)**		
	<25,000	16 (28)
	25,000-<50,000	10 (18)
	50,000-<75,000	8 (14)
	≥75,000	11 (19)
	Unknown	12 (21)

**Table 3:** Technological characteristics of focus group participants (N=57).

Characteristic		Participants, n (%)
**Has the following**		
	Cell phone	56 (98)
	Smartphone	46 (81)
	Desktop or laptop computer with internet access	35 (61)
	Tablet (eg, iPad [Apple Inc] or Kindle [Amazon])	26 (46)
**Sends or receives email**		
	At least once a day	33 (58)
	At least once a week	6 (11)
	At least once a month	5 (9)
	Less often	9 (16)
	Missing	4 (7)
**Sends or receives text messages**		
	At least once a day	39 (68)
	At least once a week	14 (25)
	At least once a month	1 (2)
	Less often	1 (2)
	Missing	2 (4)
**Accesses internet**		
	At least once a day	36 (63)
	At least once a week	7 (12)
	At least once a month	2 (4)
	Less often	9 (16)
	Missing	3 (5)
**Visits social networking sites**		
	At least once a day	24 (42)
	At least once a week	7 (12)
	At least once a month	2 (4)
	Less often	20 (35)
	Missing	4 (7)
**Uses instant messaging**		
	At least once a day	15 (26)
	At least once a week	5 (9)
	At least once a month	4 (7)
	Less often	26 (46)
	Missing	7 (12)

### Themes

The analysis of the focus group discussions revealed 5 major themes that reflected cancer survivors’ views on a web-based healthy lifestyle program related to (1) program content, (2) program design and delivery, (3) program participation, (4) technology training, and (5) receiving feedback. These themes, with related subthemes and illustrative quotes, are presented in Table 4.

**Figure 1:**
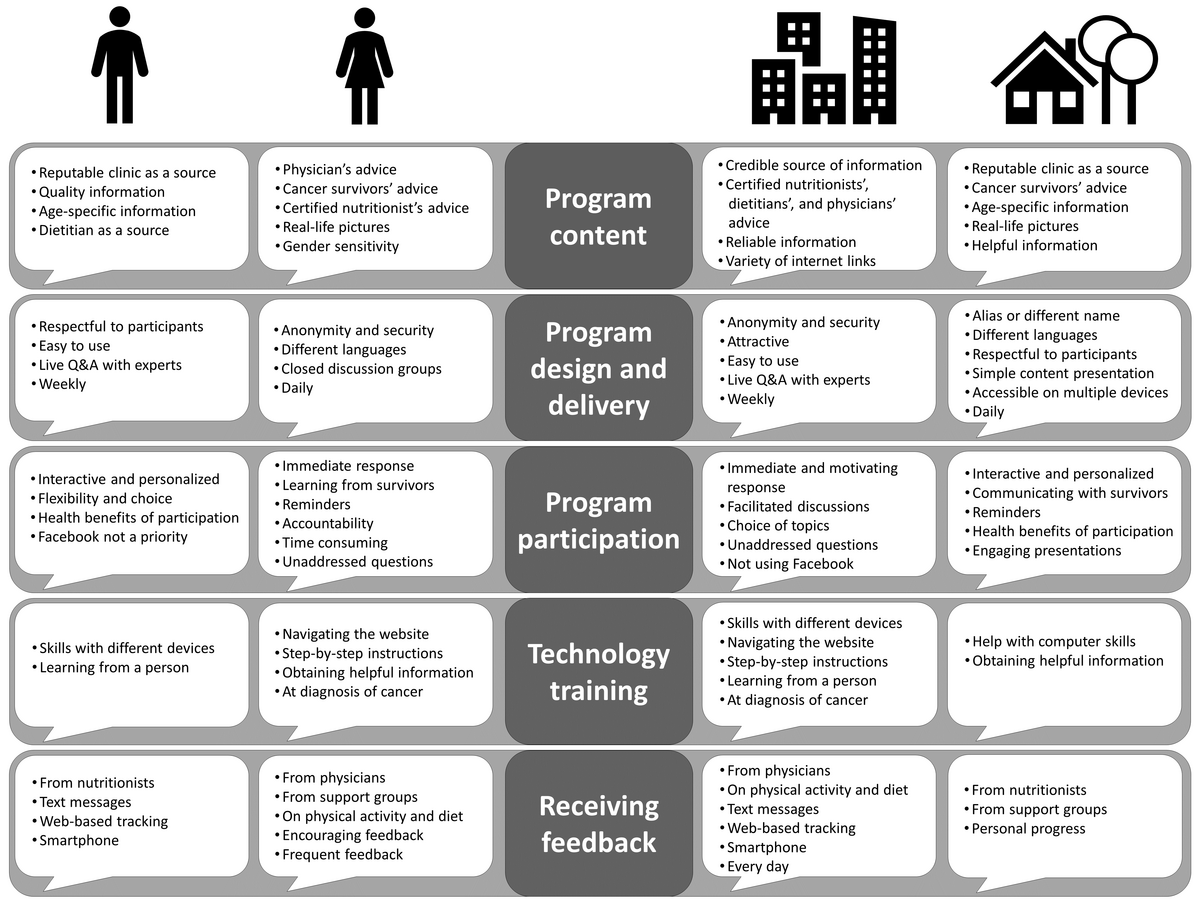
Perspectives by gender (male and female) and residence (urban and rural). Q&A: question and answer.

Using content analysis, we summarized cancer survivors’ dominant perspectives on the 3 program features (live web chat, Facebook discussion group, and e-learning sessions) by program content, design, delivery, and participation in Table 5. We also captured variations in survivors’ views by gender and rural and urban status, as shown in Figure 1.

**Table 4:** Themes, subthemes, and illustrative quotes.

Themes and subthemes			Quotes
**Program content**			
	Credibility	“I think that, you know, all the information tools that’s out there, all the resources even the live web chat that I really like, uh, because I like Facebook. So, I think all of them play a role that cancer survivors can use. If the resources and the information that’s given is valid, then I don’t have a problem with it.” [female, urban]	
	Source of information	“I don’t think any media person or, but the person should be expert in nutrition as well as the expert should have some expertise or knowledge in the disease, for example cancer. That person can give a good answer which is passing through cancer, or treatment, or maybe physician, as well as have knowledge of nutrition science.” [male, urban]	
	Information type and format	“But to have the, the video there of how certain things that would be done in exercise and uh, if you’ve got a disability here, what type of exercises I can do. I believe that it’d be very helpful for the viewer and the people that’s having discussion...people that’s uh, are cancer survivors they need to know and see examples of specific exercises they might be able to do with their various limitations, you know, because many of them are limited in this area, and that area.” [female, urban]	
**Program design and delivery**			
	Security	“I mean with privacy now in the medical field you have to be so careful. And a lot of people really are very private about their health issues. I would hate to see them miss out on this because they, everybody can see exactly who they are. I mean I know on Facebook you can create all different kinds of accounts and things. I can’t. But with something like this I think it would be kind of important maybe for it to the privacy issues to be considered in setting it up.” [female, rural]	
	Usability	“It should be easy to use. -- If you could drill down through it pretty quick, and you could just get to what you’re looking for. You know, I mean it could be this exercise side or the diet side or you know, certain based on where you’re located, something like that, and make it quickly narrow.” [female, urban]	
	Frequency	“Is that important to you that this a scheduled time thing?...Probably so. It might be a variation of times during the day at a certain time because you could plan. You know, things happen and if you miss 1 and 2 o’clock, catch one at 6 or whatever.” [male, rural]	
	Length	“So, I would say what you consider the attention span. The sense, to me, if it’s live and it’s 10 to 15 minutes, you’re going to get me 100%.” [male, rural]	
**Program participation**			
	Pros	“But the fact of the support group in discussion in a sense is that it’s...there’s other people like me that are going through what I went through or that could take advantage of what I went through and what I’m doing.” [male, rural]	
	Cons	“I probably need this program we’re talking now. I’m just illiterate with, as far as, computer illiterate, okay.” [female, urban]	
**Technology training**			
	Computer skills	“I mean if we’re trying to reach people that’s not, only knows how, that’s the only way to do it, that they might be...I mean if they already know how to navigate, all you got to do is say, ‘Here’s your program. Here’s your website’ and you’ll do it. If that’s not the case, you’re going to have to visualize it, show them. Not tell them, show them. Like you said, show me how to do it.” [female, rural]	
	Venue	“...you should be able to direct them to a class—where they are teaching people about the computer no matter what their age is, because I know there are people that are doing that at the hospital. So, if you get them on the front end and they can start then taking computer classes, then they can help themselves by knowing how to go on the internet.” [female, urban]	
	Motivation	“...as soon as a newly diagnosed person comes in, if they [doctors] know that they can follow up on the internet with certain programs, and they tell you that they are not computer literate, then you should be able to direct them to a class.”[female, urban]	
**Receiving feedback**			
	Feedback type	“You get your answer if you have a question about a certain food or type of food. You could incorporate it right away instead of having to wait.” [female, rural]	
	Occurrence	“I have to have every day here otherwise I won’t walk. Yea, I have to get on my app every day and, ‘oh my lord, I got to go walk’ kind of thing.” [female, urban]	
	Mode	“I think feedback is, is great and, and if it was me, you know, social media is, is, is, is great.” [male, urban]	
	Tracking	“It probably be usually online. Cause I’ve tried to track it on paper. Uh, cause I, I’d gotten, uh, diabetes**,** trying to figure out, keep up with what you eat.” [female, urban]	

**Table 5:** Dominant perspectives on internet program features^a^.

Themes and subthemes			Internet program features				
			Live Web Chat		Facebook discussion group		e-Learning sessions (Articulate Storyline)
**Program content**							
	Credibility	Reliable information Credible source of information		Reliable information		Relevant information	
	Source of information	Physician Certified nutritionist		Health care professional		Competent person	
	Information type and format	Being able to choose a topic Opportunity to generate further questions Communicate with others		Healthy eating and physical activity Facilitated discussion Get answers to questions Health information videos		Personalized information Interactive Using video and pictures Links to website	
**Program design and delivery**							
	Security	Anonymity		Closed group Different names		N/A^b^	
	Usability	Easily accessible		Easy to use		Simple to use Animation	
	Frequency	Every day Weekly		On a regular basis		Once a week	
	Length	15-30 minutes Up to 60 minutes		5-10 minutes 15-30 minutes		15-30 minutes	
**Program participation**							
	Pros	Expert response Flexibility and choice		Facilitated discussions Communicating with others		Customized Motivational Flexible schedule	
	Cons	Unreliable and irrelevant information Unaddressed questions Lack of experience with web chat Lack of computer skills		Not using Facebook No anonymity Lack of time Questionable quality of information		Lack of computer skills Time consuming	

^a^This table summarizes the frequent perspectives based on content analysis. See text for more perspectives.

^b^N/A: not applicable.

### Program Content

Focus group participants noted that a web-based healthy lifestyle program for cancer survivors must contain credible, high-quality, and individually tailored information developed or recommended by content experts or health care professionals. Three subthemes emerged related to program credibility, source of information, and its type and format (Table 4). All survivors were equally concerned about receiving conflicting information or information of questionable quality. A male participant observed, “...it should have information that’s credible that you trust.” Overall, urban survivors expressed more concerns about the credibility of web-based health-related information than rural survivors. The desire to receive relevant and credible information was particularly prominent in the discussion of the web-based program features (Table 5). For example, when reacting to a demonstration of the live web chat, an urban male participant stated, “I think it would be very important to make sure whoever the cancer survivor sees offering advice or providing feedback has credibility.” Both rural and male survivors were more reluctant to receive information via a Facebook discussion group because of its questionable quality:

It’s like if somebody says, “That might not be such a good idea if you try that for your health.” Or, something to guide the information that other people get because people enjoy messing up people or something.

With respect to the source of the information, survivors expressed trust in physicians, registered dietitians, and other health care professionals to guide them in the choice of healthy behaviors (Table 5). A female participant commented as follows when discussing a live web chat:

Well, first of all, you have someone that’s very knowledgeable because she’s a doctor, right? And so, we can pretty much believe what she’s gonna tell us. And she’s speaking about some very important things for all of us to know, cancer-fighting foods and how we can incorporate that into our meals every day.

Women and urban survivors were more likely to see physicians as a trustworthy information source, “I would like for it to be a physician, and I would like for it to be reputable.” Women noted that they preferred physicians’ recommendations because they had knowledge and understanding of the survivorship process:

...if there was a health care provider, someone who...knows all about cancer and knows what’s procedure and they know everything that is going on with a person in that cancer field.

Female and rural participants were also receptive to guidance from other cancer survivors in a live web chat or Facebook discussion group:

I would love to have internet live chat there with a cancer survivor. That way I can learn how to eat healthy.

Regarding healthy eating, male and urban participants were more inclined to get advice from a certified expert:

There’s so much on food out there and so many times that somebody with their plan...for healthy food that you don’t know. I would want somebody who has medical and nutritional expertise so that I could put my trust.

Cancer survivors wanted to receive information that was tailored to their needs, health conditions, and age. They particularly liked personalized health education delivered through e-learning sessions, which also allowed private interaction with the content (Table 5). A male survivor observed as follows:

It’s customized to each individual person and looks private, right? It’s just you and the interactive tool here. You plug in the information that gives it directly to you. There’s no onlookers, there’s no chat room. And you get a customized individual answer to your specific situation and the type of cancer you have, your age, all that is, like I said, is confidential, it’s private. That’s perfectly fine.

More male and urban participants talked about the need to receive information adapted to their cancer, whereas more rural survivors were interested in the information tailored to a specific age group, “...it would be satisfying that you can get right to the information for your particular age and other factors.” Women liked a program that used health information videos and pictures as visual reinforcement, particularly when introducing types of physical activity (Table 5). One woman noted when discussing e-learning sessions, “And then give maybe video, real person videos of those 5 exercises, and personalize it to a much higher degree...” Female survivors also noted the importance of being sensitive to the information presented to them, “Don’t let it tell us that we’re fat.”

Thus, a web-based healthy lifestyle program should contain information that cancer survivors find trustworthy, reliable, and tailored to their health needs, cancer type, and age. Urban survivors tended to be more concerned about information credibility and were more likely to see physicians as trustworthy information sources. Women were more inclined to receive information from a physician, whereas men preferred obtaining advice from a broader spectrum of certified experts. Women also preferred more visual reinforcements for health information and were more open to participate in Facebook discussion groups.

### Program Design and Delivery

Security, usability, frequency, and length emerged as important subthemes in the discussions of the internet program features (Table 4). Focus group participants expressed concerns about privacy issues related to participation in live web chats and Facebook discussion groups (Table 5). Regarding Facebook, a female survivor explained as follows:

I wouldn’t like it for the reason there’s no anonymity. I might not want everyone to know who I am when I am asking these questions because some people don’t want the world to know that they have cancer.

Rural participants were less concerned about privacy and suggested using different names or aliases for anonymity:

I’m very open about my cancer and a lot of people aren’t though. They’re more private and so I’m thinking they might...can they log in and do they create their own name when they log into something like this? So, like use an alias?

Although lack of anonymity was a common concern, interactions with other survivors in closed and password-protected groups were considered acceptable. Female participants particularly noted the advantages of small groups where members knew each other and could interact more freely:

Well, it’s probably better with a closed group with invitation only; that’s a small group, and then you get used to that group. And you’re familiar with everyone in that group, it will be better that way to me.

Program usability was another important consideration for cancer survivors. They wanted a web-based program to be simple, easy to use, and accessible via different devices. A male participant emphasized these features combined with the quality of the information as a condition for joining the program:

...it should be easily accessible. It should be easy to use. And it should have information that’s credible that you trust. And, I think if you have all those three,...you’re fairly likely to use it...

Female and rural participants particularly wanted the program to be simple enough for cancer survivors who had to deal with health issues on a daily basis:

You got to remember whoever is in on this going to that site, we’re dealing with the cancer and that’s a load. So, you need it simple, not because we’re ignorant on that particular stuff. We need it easy where we can just go in...

Participants noted the benefits of e-learning sessions, which use visuals and animations to make it easier for cancer survivors to understand and use the information:

...it would have to be animated if it was talking about physical exercise. If you wanted to tell them what to do that’s one thing, but it has to be animated to actually show them how to do it correctly.

In addition, the ability to ask questions and get answers emerged as an important design feature, particularly for male and urban survivors, “...a site where you can ask questions and get answers, I think all that’s wonderful, once again, I would be open to the idea.” Female participants were more interested in receiving immediate feedback so that they could use the information for their needs:

You get your answer if you have a question about a certain food or type of food. You could incorporate it right away instead of having to wait.

Although some participants did not use Facebook, they acknowledged the opportunities it offered for facilitated discussions about cancer-related issues.

The focus group participants offered varied perspectives on the frequency and duration of the program activities. Many participants believed that the weekly use of a live web chat and e-learning sessions would meet cancer survivors’ expectations. However, more rural participants wanted to engage with the program features daily, “I’d be there every day almost probably.” In general, women were willing to spend more time on program activities than men. Participants believed that spending 15 to 30 minutes on average in a live web chat and e-learning session would be ideal; however, they wanted to devote less time to participate in a Facebook discussion group, except for rural survivors, who were eager to interact with group members longer. An urban participant observed as follows:

It depends on the questions of the person, and depends on the time, availability of time with the expert who is responding, but at least five to 10 minutes are more than sufficient for any patient survivor...So, not more than 10 minutes.

Therefore, a web-based healthy lifestyle program should guarantee cancer survivors’ privacy and security, particularly in Facebook discussion groups. Rural survivors were more accepting of group interactions using aliases, whereas women saw the advantages of small closed groups. The program should be easy to use and accessible from different devices and use visuals and animations to reinforce information understanding. Participants had varied views on the frequency and length of each program feature, with women being willing to spend more time on program activities and rural survivors wanting to engage in group discussions longer.

### Program Participation

The focus group participants shared their views regarding the pros and cons of the discussed program features and their potential influence on cancer survivors’ participation in a web-based healthy lifestyle program (Table 4). Learning from experts, attractive design, flexibility, and opportunities to interact with other survivors were cited as important factors in promoting program participation. Participants liked the e-learning session feature for its flexible schedule and ability to return to the session at any time (Table 5). A male survivor observed, “One thing about the program such as this, you can go to it any time you want to.” The interactive and personalized nature of e-learning sessions was also noted as a strong appealing feature, particularly by male survivors, “Well, it’s interactive and more like a guided tour.”

At the same time, survivors appreciated the opportunity to receive an expert response to their questions in a live web chat, but noted the constraints of real-time streaming. A female participant shared, “...it would be nice that you had several choices and not miss it because you can’t be there at that time at that moment, but then would it be live?” Participation in facilitated discussions in Facebook closed groups and learning about other survivors’ experiences was also considered an appealing feature, particularly by women and rural participants:

...hearing from other people that might have had, you know, say they were taking a treatment, or they, while they were recovering,...went through similar to what I went through and certain foods helped them. It would help me, I think, to try even if I haven’t tried that food because I know that somebody has already been there.

Computer literacy was perceived as an important consideration for cancer survivors’ participation in web-based programs. While acknowledging the advantages of internet programs, participants expressed concerns about limited computer skills. Urban participants were particularly concerned about lack of experience with a live web chat:

I’m not really a computer person. So, I know I wouldn’t do that.

Similarly, survivors had little experience participating in Facebook discussion forums and felt that Facebook was “not a priority.” A male participant observed, “I wouldn’t do the group discussion on there because I don’t do Facebook and I don’t do chats.” In addition, privacy issues and lack of anonymity were perceived as barriers to participation in a Facebook group. Some women felt uncomfortable participating in a live web chat because they were afraid that their questions would not be answered:

When you are on a live chat, there is a delay. When you’re typing your question, there is a delay before it actually gets to that person. If somebody else’s question gets ahead of you, sometimes they can get caught up in the explanation for that particular person and then your question might get skipped over because somebody else is typing in also and they just, they might overlook it...I don’t like to be overlooked even though there is a delay, I still want my question answered.

Women also felt that using an e-learning session might be time consuming, despite its obvious advantages:

...I don’t have time to just be looking at that all the time...But I think it’s great. It keeps you on your toes.

Therefore, to promote cancer survivors’ participation, a web-based healthy lifestyle program should have an attractive design, provide opportunities to learn from experts, and facilitate interactions among program participants. Preference was given to e-learning sessions for their interactive and personalized nature and the ability to participate in nonreal time; however, women perceived them to be more time consuming than live interactions. Women and rural survivors tended to value Facebook closed-group discussions to learn from other cancer survivors. Computer literacy and privacy issues were perceived as barriers to program participation.

### Technology Training

Focus group participants shared their views on receiving training in computer skills and what might motivate them to consider such training (Table 4). More rural survivors acknowledged the need to learn how to use a computer, “*...*some of them may not know how to get on a computer, so they going to need to know that.” Moreover, this training should begin early on, that is, simultaneously with cancer diagnosis. A female participant observed as follows:

Like, once a person is diagnosed, tell me, okay we’ve got this wonderful tool, and this is how you use it. At least give me the option of using it whether I accept it or not, but at least put it as part of the basic plan when I am first diagnosed.

Female participants also indicated the importance of educating cancer survivors on how to navigate the website and how to use its features:

None of the tools will work if people are not educated, okay. So, we can talk about all these great things and these great ideas but until we sit people down and say, Okay, this is how you do this, this, this.

Participants suggested several venues to provide computer training for cancer survivors, including offering computer classes at a clinic for groups of newly diagnosed patients. A male survivor explained as follows:

You can set up some classes at a particular location like the... Clinic...where they would periodically teach you...how to handle access the computer. And I think that would have some merit.

Many participants preferred individualized help to standardized computer classes and recommended using support from family members and librarians. An urban survivor observed as follows:

The smartest people I ever met in life is at the library...They’ll sit down with you and show you how to go through the computer whatever.

Participants also agreed that health care providers and patients could play an important role in motivating survivors to learn computer skills:

...how do you think cancer survivors would prefer to learn about how to use internet programs and technology?...The doctors can get us started, I think for most of us.

Thus, a web-based healthy lifestyle program requires basic computer literacy, particularly among rural cancer survivors. Women were more interested in learning how to navigate the program features. Urban and male survivors preferred individualized assistance to standardized computer classes. Training should start at cancer diagnosis and be endorsed by physicians and other cancer survivors.

### Receiving Feedback

The participants acknowledged the importance of feedback to encourage cancer survivors’ participation. They provided suggestions about the feedback type, frequency, mode of delivery, and methods of diet and activity tracking (Table 4). There were variations in the frequency and type of feedback about goal achievement. Women and urban participants mentioned an interest in obtaining more specific feedback about their physical activity and healthy eating:

If you could click through to, here’s the exercises, did you do any of these? And you could just click it, boom, boom and be done. Or I ate these things and click, click and then it could send you, you came three times this week and you did these many exercises. That’d be kinda cool.

Rural participants valued feedback about their personal progress to improve accountability:

...you need something to keep you accountable,...something like this that shows you that you are overweight,...you need to exercise and...you need to eat right.

Women were more likely to use feedback as a form of encouragement to reach the goals:

I want them to continue giving me some encouragement words and say, okay, you’re doing good, you’re doing great. Keep it up.

Women were also interested in receiving immediate or frequent feedback to stay motivated:

It should be immediate response. It’s important to me to have some.

However, when speaking about losing weight, participants preferred to check their progress on a weekly basis. An urban participant explained the following:

So, I already know what I weighed before I started but at the end of the week, I need you to tell me, okay you’ve accomplished your goal, or you missed the mark. So, but only once a week for me because I can’t accomplish everything,...it takes time and with our bodies, to lose weight it’s going to take more time. So, I want to know my results by the week.

Women were more inclined to receive feedback via Facebook support groups, where members can discuss their progress:

...you had someone kind of cheering you on, but you are getting some feedback.

Men preferred text messaging to group discussions because it was simpler and convenient:

Text messages would be good because most phones now you can...they ask you what you want to do. They’re going to read the message to you. They will make it easier.

Participants noted that tracking progress could be done via internet, phone, and journaling, depending on survivors’ preferences:

...you have both options: you can write down all those foods you eat, or you can just key it into the system. So, that keying in worked for me better. I just like the computer so that works better for me.

Hence, regular feedback about cancer survivors’ progress is an important feature of a web-based healthy lifestyle program. Women and urban participants valued more specific feedback related to program activities, whereas rural survivors wanted feedback for accountability. Women were more interested in receiving immediate or frequent feedback for motivation and were more inclined to receive feedback via Facebook support groups. Men preferred text messaging and smartphones as a means to deliver feedback.

## Discussion

### Principal Findings

This qualitative study is one of the first to explore the perspectives of middle-aged and older cancer survivors on the design of a web-based healthy lifestyle intervention. Using focus group discussions with a diverse sample of 57 male and female cancer survivors from rural and urban settings, we captured a variety of perspectives related to program content, design, delivery, participation, technology training, and feedback. Participants emphasized the quality of information, participants’ privacy, ease of use, attractive design, timely feedback, and importance of considering the role of health care providers and content experts when designing web-based healthy lifestyle interventions for middle-aged and older cancer survivors. Although these themes were common across all survivors, we noted variations in views on internet program features across male and female and urban and rural participants, which may influence cancer survivors’ participation in web-based healthy lifestyle programs.

Participants reported mixed perspectives on the features requiring more staff contact (ie, live web chat and Facebook moderation by an expert) rather than interactive e-learning sessions. Although e-learning sessions are not able to provide answers to open-ended questions or allow direct, bidirectional communication with other cancer survivors or an expert, the e-learning sessions were viewed positively by our participants and can provide several additional preferred qualities (eg, tailoring, interactive, private, and more participant control of time and frequency). Once developed, such computer-based approaches require less ongoing staff contact and may be more sustainable.

Our results also emphasize the importance of a trusted, reliable source (eg, physicians); however, physicians often do not have the required training to provide the detailed diet and exercise information needed by cancer survivors [38,39]. This suggests that content experts (eg, kinesiologists, registered dietitians, etc) along with health care providers should contribute to content development when using internet technologies to promote healthy lifestyles. Finally, physicians could motivate and connect middle-aged and older cancer survivors to these resources.

In addition, lack of technology expertise is a major barrier to participating in and, thus, benefiting from internet programs that promote healthy lifestyles. Although our participants requested more staff-intensive training options, low-cost and distributable approaches to increasing technology use comfort and competence are needed.

### Strengths and Limitations

In a recent systematic review of studies on eHealth views in populations other than cancer survivors, similar themes to those that emerged in this study were reported (eg, usability, privacy, information reliability, etc) [19], thereby corroborating our results. Moreover, consistent with Rogers' DOI Theory [21], our qualitative results provide insights into important characteristics of eHealth innovations that are likely to increase diffusion (or adoption and use) of a web-based healthy lifestyle intervention by middle-aged and older cancer survivors, namely, receiving reliable and motivational information from an expert (relative advantage), personalized and relevant information, timely and frequent feedback (compatibility), ease of use, interactive and visual (complexity), computer skills training and website navigation (triability), and experiencing health benefits (observability). Future research is needed to examine other aspects of Rogers' DOI Theory that influence the adoption of an innovation, such as the characteristics of the adopter, social system, individual adoption process, and diffusion system.

Importantly, our findings extend the published literature in several ways. The majority of our participants are older cancer survivors who are rarely been studied but have reported different perspectives on eHealth when compared with older individuals without a history of cancer [18,19]. Further, we add to the gap in the literature by showing how cancer survivors’ perspectives may differ based on gender and rural and urban status [19]. In addition, technology-based interventions have been evaluated in cancer survivors as a whole, but less is known about how older cancer survivors view specific features used in developing internet approaches to promote healthy behavior change [40-43]. These strengths combined with our diverse sample (ie, 29/57, 51% women; 23/57, 40% Black survivors; 22/57, 39% without a computer with internet access; 19/57, 33% rural; 19/57, 33% with ≤12 years of education; and 16/57, 28% reporting annual household income <US $25,000 per year) and rigorous qualitative analysis have yielded unique, varied, and important insights into eHealth perspectives that are useful for others planning to use internet technologies to promote healthy lifestyles among middle-aged and older cancer survivors.

Despite its merits, our study has potential limitations. First, we did not assess other potentially important features, such as noninteractive videos and social media approaches other than Facebook. Our findings also suggest gender and urban and rural variations in views on eHealth; however, further research is needed to confirm and quantify possible differences. Moreover, it was not feasible to recruit enough cancer survivors based on age distribution without losing our rural and urban focus group stratification; therefore, we were not able to differentiate participants’ perspectives based on age groups. In contrast, our inclusion of participants regardless of their use or ownership of a computer or smartphone yielded helpful perspectives about technology training that may support middle-aged and older cancer survivors less likely to feel comfortable with technology. Owing to our study criteria, our sample included cancer survivors who were overweight or obese, were not regular exercisers, and did not eat at least 2.5 cups of fruits and vegetables daily. A recent analysis of 3367 racially and ethnically diverse cancer survivors identified through the National Health Interview Survey indicated that approximately 70% of survivors were overweight or obese and over 80% did not meet the guidelines for physical activity or fruit and vegetable consumption. Thus, our sample is likely representative of the majority of cancer survivors in the general population [44]. However, the perspectives expressed here may not be applicable to cancer survivors with advanced cancer or a cancer type with poorer prognosis nor to individuals who are non-English speaking or have at least an eighth-grade education. Finally, our participants were limited to the southeastern US state, thereby potentially reducing generalizability to other regions. Notably, this is offset by the significance of targeting a region (ie, southeastern United States) with the highest cancer mortality and comorbidity (eg, diabetes) rates in the United States [45,46].

### Conclusions

This study highlights the value of designing web-based approaches that individualize information and allow users more flexibility regarding the timing and frequency of participation. In addition, our results have several important implications. Our findings can be used to enhance the design of web-based features and educational materials used as part of providing blended care for oncology patients, an increasingly prevalent patient care paradigm that combines in-person with technology-based approaches [47-49]. Further research is needed to determine how to best connect health care providers to the information, tools, and workflows needed to encourage cancer survivor intervention participation [50]. Similarly, developing and testing strategies that increase technology comfort and competence are critical for ensuring that as many middle-aged and older cancer survivors as possible can experience the health and well-being benefits of web-based healthy lifestyle interventions now and as they age into this age category.

## Abbreviations

DOI: Diffusion of Innovation
